# Development and validation of a generalisable machine learning algorithm for identifying interstitial lung disease cohorts: a retrospective cohort study

**DOI:** 10.1016/j.eclinm.2026.103790

**Published:** 2026-02-21

**Authors:** Erica Farrand, Augustine Chung, Jisha Joshua, Huawei Dong, Hunter Mills, Albert Lee, Martin Ieong, Lakshmi Radhakrishnan, Oksana Gologorskaya, Atul Butte

**Affiliations:** a Department of Medicine, University of California San Francisco, San Francisco, CA, USA; b Department of Medicine, University of California Los Angeles, Los Angeles, CA, USA; c Department of Medicine, University of California San Diego, San Diego, CA, USA; d Department of Medicine, University of California Irvine, Irvine, CA, USA; e Bakar Computational Health Sciences Institute, University of California San Francisco, San Francisco, CA, USA; f Academic Research Services, Information Technology, University of California, San Francisco, CA, USA

**Keywords:** Interstitial lung disease, Machine learning, Artificial intelligence, Real-world-data, Data science

## Abstract

**Background:**

Large electronic databases are powerful tools for studying rare diseases, however accurate Interstitial Lung Disease (ILD) classification remains challenging. Rule-based approaches rely heavily on diagnostic codes—unreliable markers of ILD. We aimed to develop and externally validate an ILD classification algorithm that robustly identifies prevalent cases using routinely captured electronic health record (EHR) data.

**Methods:**

In this retrospective model development and validation study, we used EHR data from the UC Health Data Warehouse, a multi-institutional dataset from six academic centres in California, USA (2012–2024). Data from individuals ≥18 years with ≥ five encounters were included. We developed the Universal ILD Classifier, a machine learning model developed on standardised EHR data elements from UC San Francisco (January 1, 1981–January 6, 2025). The algorithm was converted to an EHR-agnostic common data model, to enable external validation across three independent sites (UC Irvine, Los Angeles, and San Diego; January 1, 2012–April 30, 2025). Features included diagnostic and procedure codes, laboratory results, medications, demographics, and utilisation metrics. The main outcome was algorithm performance assessed by positive predictive value (PPV), sensitivity, F1-score, and receiver operative characteristic–area under the curve (ROC-AUC). Performance was compared with two widely used rule-based classification methods.

**Findings:**

The Universal ILD Classifier, developed on data from 203,976 patients and validated on data at three independent sites (N = 250 per site), demonstrated robust generalisability, achieving average PPV = 0.67 (0.58–0.72), sensitivity = 0.97 (0.94–0.99), F1-score = 0.79 (0.72–0.84), and ROC-AUC = 0.96 (0.94–0.97). It consistently outperformed both rule-based methods, which had PPVs = 0.55 (0.50–0.59) and 0.67 (0.61–0.73), sensitivities = 0.98 (0.96–0.99) and 0.59 (0.53–0.64), F1-scores = 0.71 (0.66–0.74) and 0.63 (0.57–0.68), and ROC-AUCs = 0.80 (0.78–0.82) and 0.73 (0.70–0.76) respectively.

**Interpretation:**

Accurate patient identification is essential for epidemiological studies and ILD clinical trials. The Universal ILD Classifier leverages commonly available EHR data and outperforms rule-based approaches, supporting more reliable large-scale ILD research and offering a foundation for further refinement with additional features. Limitations inherent to retrospective EHR analyses, including misclassification, residual confounding, and limited generalisability, may have influenced effect estimates and should be considered when interpreting these findings.

**Funding:**

10.13039/100001003Boehringer Ingelheim Pharmaceuticals, Inc. (BIPI).

**Research in context**

**Evidence before this study**

We searched PubMed from inception to July 31, 2024 for studies evaluating automated or algorithmic identification of Interstitial Lung Disease (ILD) using large electronic datasets using search terms including interstitial lung disease, ILD, pulmonary fibrosis, electronic health record (EHR), claims data, real-world data, and reviewed reference lists of retrieved papers. Most identified approaches were rule-based algorithms reliant on diagnostic codes, which frequently showed substantial misclassification. Where performance appeared strong, it often reflected institution-specific coding practices or use within ILD subtypes (e.g. rheumatoid arthritis ILD) where codes are more reliable. Only a small number of machine learning (ML) studies were identified, nearly all single-centre, with limited external validation, and non-harmonised data structures necessary for generalisability. No prior study reported an EHR-agnostic, standardised ML model validated across multiple independent health systems.

**Added value of this study**

This study introduces the Universal ILD Classifier, the first ML algorithm trained on a common data model and externally validated across multiple health systems. Using harmonised, high-dimensional clinical features—including diagnoses, procedures, laboratory values, medications, and utilisation—the classifier achieved higher positive predictive value, sensitivity, F1-score, and ROC-AUC than two widely used rule-based methods. These findings show that standardised ML phenotyping can overcome limitations of diagnostic code-based ILD identification and can be deployed across institutions without site-specific re-programming.

**Implications of all the available evidence**

Multi-institutional, EHR-agnostic ML algorithms substantially improve ILD cohort identification, a major bottleneck to epidemiological studies, real-world evidence generation, and clinical trial recruitment. The Universal ILD classifier provides a scalable, generalisable alternative to rule-based algorithms through explainable AI. However, variability in EHR data completeness, reliance on structured data only, and validation limited to academic centres highlight areas requiring further evaluation.

## Introduction

Machine learning (ML), a branch of artificial intelligence (AI), has revolutionised health research by applying advanced statistical and computational methods to analyse large datasets and emulate human learning.^1^^,^^2^ ML algorithms have supported predictive modelling across numerous conditions, enhancing diagnostic accuracy, streamlining workflows, and improving patient outcomes.^3^^,^^4^

ML techniques are particularly valuable in rare disease research, where diagnoses rely on a complex combination of clinical, imaging, pathology, and laboratory data to make diagnoses that are often incompletely or inaccurately captured by diagnostic codes. Manual data review to build rare disease research cohorts is labour-intensive and limits study size. By identifying patterns in large electronic datasets, ML can generate more precise and scalable electronic disease signatures support cohort construction.

A major challenge is that many ML algorithms function as “black boxes” limiting interpretability, trust, and assessment of error or bias, and ultimately constrains their application.^5^^,^^6^ Explainable AI provides human interpretable insights through model simplification, feature importance analyses, and visualisation techniques that clarify model reasoning and promote real-world acceptance.^7^, ^8^, ^9^

In this study, we tested and validated an explainable ML algorithm for interstitial lung disease (ILD). Traditional ILD cohort identification relies on a labour-intensive manual review process which often limits the number of patients who can be screened and constrains final cohort sizes.^10^, ^11^, ^12^, ^13^, ^14^ Alternatively, rule-based approaches, dependent on ILD diagnostic codes, automate ILD cohort identification. While rule-based approaches can perform well in settings where diagnostic coding and structured clinical documentation are accurately and consistently applied,^15^^,^^16^ in many settings ILD diagnostic codes are inconsistently applied, non-specific, or used to capture diagnostic uncertainty. This results in lower sensitivity and positive predictive value and increasing risk of both over- and under-ascertainment.^17^, ^18^, ^19^ To address these limitations, we leveraged routinely collected, high-dimensional electronic health record (EHR) data to expand the feature set beyond diagnostic codes and develop a robust explainable ILD classification algorithm. Using ILD as a model, we demonstrate how high-dimensional medical data can automate cohort identification and improve classification accuracy of rare diseases, facilitating epidemiological, observational, clinical outcomes, and health services research using large electronic datasets.

## Methods

### Internal algorithm development

The initial ILD Classification Algorithm was developed using the University of California De-identified Clinical Data Warehouse (DeID-CDW) derived from electronic health record (EHR) data on 5,528,951 patients (2012–2022).^20^ The UCSF ILD Database, a longitudinal cohort study of adult patients seen in the UCSF ILD Clinic, with a gold-standard multidisciplinary diagnosis,^21^ served as the labelled dataset for model training. Only 10–15% of this cohort were ultimately found not have ILD. We therefore enriched the training data with randomly sampled non-ILD cases from DeID CDW. Candidate enrichment sizes ranged from 2000 to 100,000; we selected an enrichment size = 10,000 to balance computational efficiency with adequate representation of the general population.

EHR data available between January 1, 1981 and January 6, 2025 was included in the training data set. Participants were included if they were ≥18 years with a minimum of five EHR encounters. This utilisation parameter was used to ensure that each participant had the minimally sufficient structured data available to construct a meaningful feature vector and support valid classification. This cohort was randomly split 80/20 into training and testing sets.

Candidate features were selected through a structured, clinically grounded process and guided by explainable AI principles (interpretability, explainability, transparency).^22^^,^^23^ Pulmonary and ILD experts reviewed all 3229 variables, identifying 334 variables with justifiable clinical or biological plausibility in ILD. ANOVA was used to further reduce dimensionality by selecting variables with strong discriminatory signal. Dimensionality reduction enhances generalisability, improves interpretability, and avoids overfitting; alternative approaches (e.g. L1-regularised logistic regression) did not meaningfully improve performance. Finally, we applied gradient boosting tree (GBT), a prediction modelling technique based on decision trees,^24^ to perform ILD classification. Key hyperparameters (maximum depth, learning rate, number of trees, and subsample size) were optimised using cross-validation within the training set. GBT natively accommodated missing values by learning default split directions, preserving clinically informative missingness patterns. Additionally, GBT performed well compared to alternative options, trained quickly on large datasets, allowed inclusion of both numerical and categorical variables, and yielded results that were interpretable and explainable to human users. For each adult patient in the training and test set, the ILD Classification Model predicted a probability of ILD between 0 and 1.

Model selection relied on the Brier score, which evaluates accuracy and calibration of probabilistic predictions. The Brier score is particularly informative in case-imbalance settings, such as classifying a rare disease within a general population, because it penalises models that are overconfident or poorly calibrated even when their discrimination appears strong. In addition, four well-established measures of model performance in a class imbalance scenario were used to assess model performance: precision, recall, F1-score, and receiver operative characteristic–area under the curve (ROC-AUC). Precision and recall are data science terms for positive predictive value (PPV) and sensitivity respectively. We will use these more familiar terms moving forward. F1-score is the harmonic mean of PPV and sensitivity and is a widely accepted metric when the objective is to minimise both false positives and false negatives. The ROC-AUC is widely used to plot the false-positive rate vs. the true-positive rate for all possible decision thresholds. A full description of the initial model development and performance was previously published.^25^ In testing, the best performing ILD Classification Model had a PPV = 0.93, sensitivity = 0.87, F1-score = 0.83 and ROC-AUC = 0.93. We also developed a modified version of the model leveraging only structured variables, as natural language processing tools to extract unstructured data from clinical notes are not yet widely available at many medical institutions. This Modified ILD Classification Algorithm had a PPV = 0.72, sensitivity = 0.82, F1-score = 0.77, and ROC-AUC = 0.85 (Supplement Table S1), as previously published.^25^

### Internal algorithm modification for general use

To enable implementation across health systems, the ILD Classification Algorithm was adapted to the Observational Medical Outcomes Partnership (OMOP) Common Data Model (CDM). OMOP CDM defines a standardised approach to organising healthcare data into a common format and vocabulary.^26^^,^^27^ Therefore, an algorithm designed to run on OMOP CDM variables would use a single unified code pipeline to format model inputs across different data resources, supporting *generalisable* and *scalable* models. OMOP organises data in relational databases consisting of structured data fields, while data elements derived from clinical notes are less commonly included^28^; therefore, the modified version of the ILD Classification Algorithm leveraging only structured data fields was mapped to standardised OMOP ontologies. This version, adapted to run on OMOP, will be referred to as the Universal ILD Classifier. It was trained on UCSF OMOP data (2012–2025). A summary of the iterative models developed in our pipeline is outlined below.

1. **ILD Classification Algorithm**–included all available institutional data, including data from clinical notes
2. **Modified ILD Classification Algorithm**—limited to structured institutional data (no variables derived from clinical notes)
3. **Universal ILD Classifier**—OMOP-based adaptation of the Modified ILD Classification Algorithm (structured institutional data mapped to standardised OMOP vocabularies)

### Model testing

The Universal ILD Classifier was trained and validated on data reserved from the initial phase of model development. For each adult patient in the UCSF training set, the universal ILD Classifier predicted a probability of ILD between 0 and 1. To assess the model's performance across the full range of ILD probabilities, the scores were grouped by decile, and 250 patients were randomly selected for outcome verification with balance across deciles. This model calibration was chosen to mitigate the risk of patients with ILD being under-represented or entirely absent in the test set, a likely occurrence in the setting of a rare disease (Supplement Figure S1) and preserve representation across prediction scores for robust model evaluation. An ILD expert (E.F.), blinded to the classifier's predictions, performed structured chart review of imaging, pathology, and clinical notes to determine the presence or absence of ILD. Expert labelling informed model performance metrics and threshold selection using iterative ROC analysis to optimise ILD classification utility in a real-world setting.

### External model validation

The universal ILD classifier was applied to de-identified OMOP datasets from January 1, 2012 to April 30, 2025 across three institutions, University of California Los Angeles (UCLA), San Diego (UCSD), and Irvine (UCI). All validation sites used the Epic EHR system (Verona, WI) and reviewed institution-level data that had been generated and stored independently based on the local Epic instance. Epic data from University of California institutions was transformed to OMOP CDM and centrally stored in the UC Health Data Warehouse (UCHDW). UCHDW is a unique data asset which contains data since 2012 on 9.4 million patients from six independent medical centres (inclusive of UCSF, UCLA, UCSD and UCI) covering all ten regions of California and reflecting regional variations in age, race, ethnicity and socioeconomic status.^29^

Consistent with testing and training, the external validation sample set was limited to information on adults ≥18 years with at least 5 encounters. The Universal ILD Classifier predicted a probability of ILD between 0 and 1 for each patient in the validation set. At each site, a validation set was randomly sampled across prediction deciles (N = 250 at each site, based on feasibility). An ILD expert at each participating validation site (A.C., J.J., H.D.) blinded to the classifier's predictions performed structured chart review to determine the presence or absence of ILD, based on documented multidisciplinary diagnosis, chest imaging reports, pathology reports, and clinical notes. Expert labelling was compared to classifier predictions to calculate model performance metrics.

### Statistical analysis and model evaluation

Considering ILD is a rare disease, classification accuracy was not a suitable measure of model performance since most patients within a general adult population will not have ILD. Therefore, we again relied on four well-established measures of model performance in a class imbalance scenario: PPV, sensitivity, F1-score, and ROC-AUC.

After externally evaluating the performance of the Universal ILD Classifier at three institutions, we compared its performance to two common rule-based approaches that used prespecified combinations of International Classification of Diseases (ICD) diagnosis and procedure codes in an if-then framework to identify ILD cohorts.^17^, ^18^, ^19^ Rule-based Approach 1 (high-sensitivity definition), adults ≥18 years of age were classified as having ILD if they had at least one ILD diagnosis code recorded in any encounter. Rule-based Approach 2 (high-PPV definition), adults ≥18 years of age were classified as having ILD if they had at least two ILD diagnosis codes separated by ≥30 days and at least one CT chest procedure code.^30^, ^31^, ^32^, ^33^, ^34^ We ran both rule-based approaches on the University of California wide dataset (UCHDW), using the same population and eligibility criteria as the Universal ILD Classifier. For each rule-based approach, performance metrics (PPV, sensitivity, F1-score, and ROC-AUC) were calculated using the same expert-labelled validation samples. We conducted a comparative analysis of the misclassification patterns made by the Universal ILD Classifier compared to the two rule-based approaches. First, we applied McNemar's test to evaluate whether there was a statistically significant difference in the misclassification rates between the Universal ILD Classifier and the rule-based approaches (i.e. does the frequency of misclassifications differ amongst methods?). We then applied Kolmogorov–Smirnov (K–S), a non-parametric test, to compare the cumulative distribution functions of the predicted probabilities for misclassified instances made by each approach (i.e. are the different methods assigning systematically different probability distributions to the predictions?). The K–S statistic (D) provides insight into whether different approaches are making misclassifications in systematically different ways as evidenced by their probability distribution, in addition to differences in the frequency of misclassification errors (McNemar's). For both tests we used a significance level of p ≤ 0.0167 based on Bonferroni correction for multiple pairwise comparisons. This study was approved by the Institutional Review Boards of UCSF (IRB # 22-37799), UCI (IRB #2406), UCLA (IRB# 22-1822), and UCSD (IRB# 806138). The requirement of informed consent was waived owing to the use of de-identified data. All methods adhered to relevant ethical guidelines and regulatory standards. Reporting followed the TRIPOD-AI (Transparent Reporting of multivariable prediction model of Individual Prognosis Or Diagnosis-Artificial Intelligence) recommendations.

### Role of the funding source

The funder of the study had no role in study design, data collection, data analysis, data interpretation, or writing of the report. Authors had full access to the study data and final responsibility for the decision to submit for publication (EF, AC, JJ, HD, HM, AL, MI, LR, OG, AB).

## Results

**Fig. 1:**
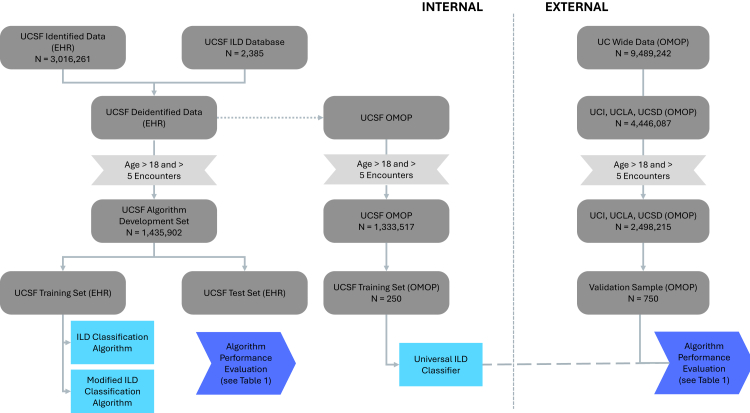
Flow diagram of train, test, and validation sets for algorithm development. Flow diagram demonstrating the creation of training, testing, and external validation cohorts for development of machine-learning classification algorithms. Data sources included the University of California San Francisco ILD Database and De-identified Clinical Data Warehouse, and University of California Health Data Warehouse and mapped Observational Medical Outcomes Partnership (OMOP) data. *UCSF, University of California San Francisco; UCI, University of California Irvine; UCLA, University of California Los Angeles; UCSD, University of California San Diego.*

The baseline characteristics of participants in the training and validation cohorts are available in Table 1. In internal model evaluation, a total of 1,333,517 patients were included in the UCSF OMOP set after excluding paediatric patients (age <18) and patients who did not have at least five encounters (Fig. 1). A previous demographic comparison between included and excluded patients showed similar distributions in age, sex, and primary insurance type.^25^ We tested the performance of Universal ILD Classifier running on OMOP CDM compared to the modified ILD Classification Algorithm yielding comparable results at UCSF, with PPV = 0.72 vs. 0.72, sensitivity = 0.99 vs. 0.82, F1-score = 0.83 vs. 0.77, and ROC-AUC = 0.95 vs. 0.85, respectively (Supplement Table S1).

**Table 1:** Participant characteristics at baseline in the derivation and validation cohorts.

	Training cohort (N = 203,976)	Validation cohort (N = 926)
Location	UCSF	UCI, UCLA, UCSD, UCSF
Age, years	65 (16.6)	51 (19.7)
Sex		
Female	480 (52%)	115,936 (57%)
Male	445 (48%)	87,715 (43%)
Unknown	1 (0%)	325 (0%)
Race		
Asian	105 (11%)	21,337 (10%)
Black	53 (6%)	12,393 (6%)
Native American or Alaska Native	4 (0%)	985 (0%)
Native Hawaiian or Pacific Islander	2 (0%)	6805 (3%)
Other	162 (18%)	26,504 (13%)
Unknown/Declined	75 (8%)	31,971 (16%)
White	525 (57%)	103,981 (52%)
Ethnicity		
Latinx	157 (17%)	22,937 (11%)
Not Latinx	679 (73%)	138,946 (68%)
Unknown	90 (10%)	42,093 (21%)

UCSF, University of California San Francisco; UCI, University of California Irvine; UCLA, University of California Los Angeles; UCSD, University of California San Diego.

**Fig. 2:**
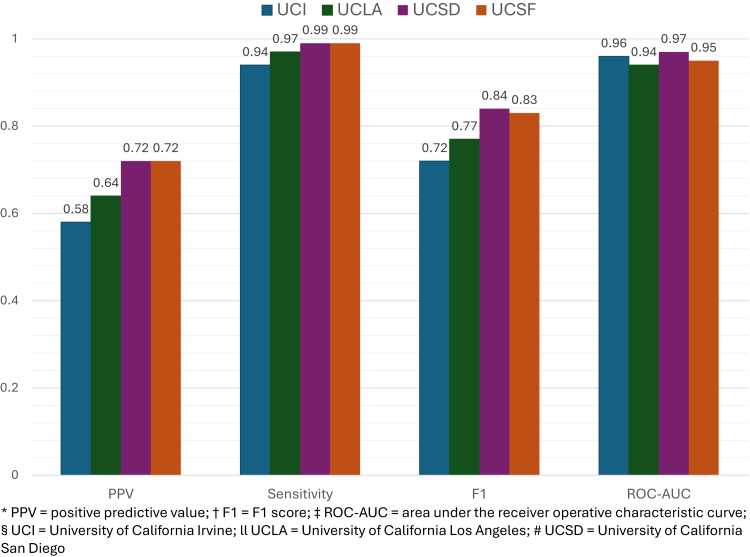
Universal ILD classifier performance across validation sites. Performance metrics for the Universal ILD classifier evaluated across four health-system datasets. Site-specific PPV, F1-score, and ROC-AUC curve are displayed. *PPV, positive predictive value; F1, F1-score; ROC-AUC, area under the receiver operating characteristic curve; UCI, University of California Irvine; UCLA, University of California Los Angeles; UCSD, University of California San Diego; UCSF, University of California San Francisco.*

Next, in external model validation, a total of 2,498,215 adult patients were identified for inclusion across all three external validation sites (UCI, UCLA, and UCSD) (Fig. 1). The Universal ILD Classifier performed well across all three sites on all performance metrics with an average PPV = 0.67 (0.58–0.72), sensitivity = 0.97 (0.94–0.99), F1-score = 0.79 (0.72–0.84), and ROC-AUC = 0.96 (0.94–0.97) (Fig. 2, Supplement Table S1).

**Fig. 3:**
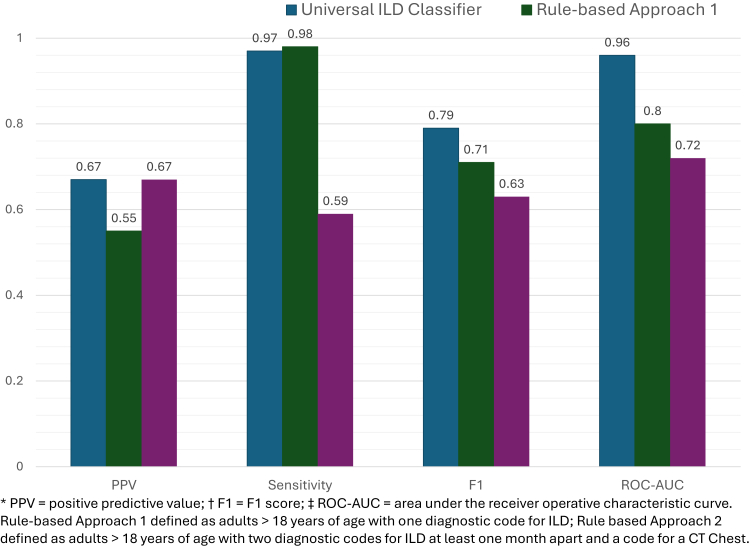
Universal ILD classifier performance compared to rule-based approaches. Comparison of Universal ILD Classier performance to two rule-based ILD identification algorithms. Metrics include positive predictive value (PPV), F1-score, and ROC-AUC. Rule-based Approach 1: adults aged ≥18 years with ≥1 diagnostic code for ILD. Rule-based Approach 2: adults aged ≥18 years with ≥2 ILD diagnostic codes at least 1 month apart plus a CT chest code. *PPV, positive predictive value; F1, F1-score; ROC-AUC, area under the receiver operating characteristic curve.*

In comparative analysis we present the data in the following order: Universal ILD Classifier, Rule-based Approach 1, Rule-based Approach 2. The Universal ILD Classifier performed most consistently across all metrics as compared to both rule-based approaches when evaluated on the same expert-labelled validation samples: PPV (0.67 vs. 0.55 and 0.67), sensitivity (0.97 vs. 0.98 and 0.59), F1-score (0.79, 0.71 and 0.63), and ROC-AUC (0.96 vs. 0.80 and 0.73) (Fig. 3). When evaluating the pattern of errors made by the Universal ILD Classifier compared to both rule-based approaches using McNemar's test and a significance level of p ≤ 0.017, we observed that the misclassification rate was significantly different (lower) for the Universal ILD Classifier compared to either Rule-based Approach 1 (p = 2.52e^−13^) or Rule-based Approach 2 (p = 0.004). However, the misclassification rates were not significantly different between to the two rule-based approaches (p = 0.03) (Table 2). We then applied the K–S test to evaluate differences in the misclassification patterns of the three methods (corrected significance value of p < 0.017), and observed that the patterns were significantly different across all three approaches, p < 2.2e^−16^ (ILD Classifier vs. Rule-based Approach 1), p < 2.2e^−16^ (ILD Classifier vs. Rule-based Approach 2), and p = 0.001 (Rule-based Approach 1 vs. Rule-based Approach 2) (Table 2). When plotting the misclassification patterns, we observed that both rule-based approaches resulted in misclassification errors at all likelihoods of ILD (i.e. classified patients with very low likelihood of ILD as positive cases and patients with very high likelihood of ILD as negative cases) (Supplement Figure S2).

**Table 2:** Model misclassifications—comparison of universal ILD classifier to rule-based approaches.

	McNemar's test χ^2^ (p-value)	Kolmogorov–Smirnov test D (p-value)
Universal ILD Classifier vs. Rule-based Approach 1	53.55 (2.52e^−13^)	0.71 (<2.2e^−16^)
Universal ILD Classifier vs. Rule-based Approach 2	12.40 (0.0004)	0.78 (<2.2e^−16^)
Rule-based Approach 1 vs. Rule-based Approach 2	4.65 (0.03)	0.14 (0.001)

Rule-based Approach 1 defined as adults ≥18 years of age with one diagnostic code for ILD; Rule based Approach 2 defined as adults ≥18 years of age with two diagnostic codes for ILD at least one month apart and a code for a CT Chest. Adjusting for multiple pairwise comparisons, p = 0.017.

## Discussion

The success of epidemiological studies, health services delivery research, and clinical trials depends on accurate cohort identification, a process that is resource-intensive, especially for rare diseases such as ILD. Using readily available high-dimensional EHR data, we demonstrate that ML can significantly improve ILD cohort identification. The Universal ILD Classifier, built upon a robust GBT algorithm and adapted to run on a CDM achieved high PPV, sensitivity, F1-score, and ROC-AUC scores in training, testing, and multi-site validation, underscoring its capability to accurately and consistently identify patients with ILD in a general adult population. Furthermore, it did so while balancing both the impact of false negatives and false positives in making predictions, providing a tool to generate retrospective cohorts of patients with ILD that represent real-world cohorts.

Compared with current rule-based approaches, the Universal ILD Classifier, demonstrated more consistent performance and significantly fewer misclassification errors. The rule-based approaches underscored greater susceptibility to coding inconsistencies and demonstrated non-systematic error patterns without identifiable trends that could be easily leveraged for future improvement. In contrast, the Universal ILD Classifier's performance reflected the advantages of ML models that integrate broader clinical data and capture complex relationships among features. It is probable that iterative advancements in data science, including targeted adjustments to the features fed into the model and/or refinements to the modelling techniques, could improve future performance of the Universal ILD Classifier. The different opportunities for performance improvement further support the benefits of adopting a ML-based approach.

A key strength of this work is the Universal ILD Classifier's generalisability, matching an advantage previously only seen with rule-based approaches. Unlike our prior ILD Classification Algorithm and other published ML ILD algorithms which were trained and tested on a single centre's data and/or developed to run on a specific EHR,^35^^,^^36^ the Universal ILD Classifier runs on a Common Data Model which is EHR-agnostic, thereby yielding a tool that is well-suited for widespread implementation and facilitation of multi-centre cohort-development. The Universal ILD Classifier not only provides a reliable and improved method for ILD cohort identification compared to rule-based approaches, but the standardised and generalisable approach allows it to be easily scaled and adapted at different institutions to construct large real-world retrospective cohorts of patients with ILD.

Several limitations warrant consideration. Variations in EHR data capture across institutions and incomplete or inconsistent variables could influence model performance, introduce bias, and impact generalisability. As the breadth and depth of EHR data expands with investments in health systems' informatics, we anticipate more uniform and improved performance. Our validation was conducted in academic medical centres. Additional evaluation in non-academic health systems is needed. Because the Universal ILD Classifier leverages structured data fields only, it does not consider valuable information present in unstructured data sources that might is enhance model performance. We recognise that extracting unstructured data from the EHR requires resources that are not currently available in most health-systems. As advanced statistical algorithms in natural language processing and large language models become more widespread and mature, we anticipate unstructured data will play a growing role in the generation of real-world evidence and be integrated into future models, driving improvement in model performance. A multidisciplinary diagnosis was used to determine the presence of ILD in the training set, where a composite of multidisciplinary documentation, chest imaging reports, pathology reports, and clinical documentation was used to determine the presence or absence of ILD in the test and validation sets. Excluding individuals with <5 encounters may reduce representation of patients with infrequent healthcare engagement. Finally, we did not apply explicit imputation, therefore differential missingness across predictors and sites could introduce bias and could affect the model's generalisability.

There was a notable improvement in sensitivity from the modified ILD Classification Algorithm to the Universal ILD Classifier (0.82 vs. 0.99). We believe the primary driver of this change was the transition to the OMOP CDM, which required comprehensive remapping of concepts and likely strengthened feature definitions while reducing misclassification arising from inconsistent local coding. Additional factors that may have affected model discrimination include differences in negative-case sampling for the two models, differences in EHR timeframes (the Universal ILD Classifier was trained on a later OMOP-mapped EHR extract that contained additional data), and minor differences in the decision threshold (the Universal ILD Classifier used a threshold selected a priori based on the F1-score within the training set, which differed slightly from the threshold used in the earlier model, selected using a heuristic approach).

We foresee multiple potential applications of automating ILD classification using ML algorithms. Using the Universal ILD Classifier to analyse large-scale real-world data from diverse healthcare settings could generate valuable insights into ILD epidemiology, treatment patterns, and patient outcomes. This evidence could in turn inform public health strategies, policy decisions, and healthcare resource allocation. The Universal ILD Classifier could be used to optimise the design and execution of clinical trials by identifying and stratifying patient cohorts based on specific disease characteristics. Incorporating the Universal ILD Classifier into clinical decision support systems could assist healthcare providers in diagnosing ILD. Expanding the use of the classifier across multiple institutions and healthcare systems could facilitate large-scale, collaborative research efforts critical to rare disease research. Additionally, the techniques and methodologies used to develop the Universal ILD Classifier could be adapted to other rare respiratory disorders, enhancing our ability to leverage real world data sources to study a range of conditions.

Beyond demonstrating the value of a universal ILD classifier, this work establishes a platform for extending EHR-based phenotyping into specific subgroups. Although the present model was intentionally trained at the aggregated ILD level to maximise sensitivity in real-world settings and generalisability across heterogeneous health systems, combined use with downstream subtype-specific classifiers and risk prediction models offers a clear roadmap for increasingly precise, adaptive and clinically actionable ILD phenotyping. Together these approaches can enable richer characterisation of ILD heterogeneity, support targeted cohort identification, and accelerate translational and clinical research across the ILD spectrum.

In summary, identifying ILD patient cohorts is an essential and resource-intensive first step in clinical research. In this study we demonstrate the promise and feasibility of ML in addressing the challenges associated with cohort identification in rare disease research. By moving beyond rule-based systems and utilising advanced algorithms that can leverage high-dimensional, readily-available data, researchers can build more accurate and representative ILD cohorts which will enhance our understanding and treatment of these complex diseases.

## Contributors

Study concept and design: E.F., A.B.

Literature search: E.F.

Data collection: E.F., A.C., J.J., H.D., H.M., L.R., O.G.

Figures: E.F., H.M., A.L.

Data analysis: E.F., H.M., M.I., L.R., O.G.

Data access and verification: E.F., M.I., L.R.

Data interpretation: E.F., A.C., J.J., H.D., H.M., A.L., M.I., L.R., O.G., A.B.

Manuscript drafting: E.F., A.C., J.J., H.D., H.M., M.I., L.R., O.G.

Critical review of manuscript for content: All authors.

Obtained funding: E.F.

## Data sharing statement

The de-identified electronic health record data used in this study are not publicly shareable owing to institutional data governance policies and patient privacy regulations. Aggregated data supporting the findings and analytic code are available from the corresponding author upon reasonable request and subject to a data use agreement.

## Declaration of interests

E.F. reports institutional support from Boehringer Ingelheim, the Doris Duke Charitable Foundation, and the Pulmonary Fibrosis Foundation related to the submitted work; additional institutional grant support from the NHLBI (K23HL175213-01), the Nina Ireland Program for Lung Health, and the Marcus Program in Precision Medicine Innovation; honoraria paid personally from the American Lung Association; and an unpaid leadership role on the Pulmonary Fibrosis Foundation Steering Committee. A.C. reports institutional support from Boehringer Ingelheim for work related to the submitted manuscript; consulting fees paid personally from the FDA Orphan Products Division for service on review committees related to ILD applications; consulting fees from MEDACorp for work with venture capital firms on ILD pipeline candidates; and honoraria from Veracyte for an internal lecture. J.J. reports grant support from Boehringer Ingelheim Pharmaceuticals, Inc. related to the submitted work. H.D. reports institutional support from Boehringer Ingelheim for work related to the submitted manuscript, and personal honoraria from Boehringer Ingelheim for educational presentations. A.B. reported grants and non-financial support from Progenity, personal fees and other from NuMedii, personal fees and other from Personalis, grants and personal fees from NIH (multiple institutes), grants from L'Oreal, grants, personal fees and non-financial support from Genentech, personal fees and non-financial support from Merck, personal fees and non-financial support from Lilly, personal fees and other from Assay Depot, personal fees and non-financial support from Geisinger Health, personal fees and other from GNS Healthcare, personal fees and other from uBiome, personal fees and non-financial support from Roche, personal fees from Wilson Sonsini Goodrich & Rosati, personal fees from Orrick, Herrington & Sutcliffe, personal fees from Verinata, personal fees from 10× Genomics, personal fees from Pathway Genomics, personal fees from Guardant Health, personal fees from Gerson Lehrman Group, personal fees and other from Nuna Health, personal fees from Samsung, personal fees and non-financial support from Milken Institute, personal fees and non-financial support from Brown University, personal fees and non-financial support from Oregon Health Sciences University Knight Cancer Centre, personal fees and non-financial support from Vermont Oxford Network, personal fees and non-financial support from University of Chicago, personal fees and non-financial support from Mount Sinai School of Medicine, personal fees and non-financial support from University of Pittsburgh School of Medicine, personal fees and non-financial support from Capital Royalty Group, personal fees and non-financial support from Champalimaud Foundation, personal fees and non-financial support from Scripps Translational Science Institute, personal fees and non-financial support from Washington University in St. Louis, personal fees and non-financial support from University of Maryland, personal fees and non-financial support from HIMSS, personal fees and non-financial support from Federation of Clinical Immunology Societies (FOCIS), personal fees and non-financial support from Kansas City Area Life Sciences Institute, personal fees and non-financial support from Association for Molecular Pathology, personal fees and non-financial support from TEDMED, personal fees and non-financial support from Physician's Education Resource, personal fees and non-financial support from Optum Labs, personal fees and non-financial support from National Jewish Health, personal fees and non-financial support from Federation of the Israeli Societies for Experimental Biology (FISEB), personal fees and non-financial support from Pfizer, personal fees and non-financial support from Bayer, personal fees and non-financial support from Fusion Conferences, personal fees and non-financial support from Accelerating Biopharmaceutical Development (AccBio), personal fees and non-financial support from Three Lakes Partners, personal fees and non-financial support from Paediatric Academic Societies, personal fees and non-financial support from Korean Society for Biochemistry and Molecular Biology, personal fees and non-financial support from Human Proteomics Organization (HUPO), personal fees and non-financial support from HudsonAlpha, personal fees and non-financial support from Tensegrity, grants from Intervalien Foundation, personal fees and non-financial support from Association for Academic Health Sciences Libraries, personal fees and non-financial support from Westat, personal fees and non-financial support from FH Foundation, personal fees and non-financial support from University of Kentucky, personal fees and non-financial support from University of Pennsylvania, personal fees and non-financial support from The Transplantation Society, grants and non-financial support from California Office of Planning and Research, personal fees and non-financial support from WuXi, personal fees and non-financial support from University of Arkansas, personal fees and non-financial support from FlareCapital, non-financial support from National Academies, personal fees and non-financial support from Helix, personal fees and non-financial support from American Urological Association, personal fees and non-financial support from Association for American Medical Colleges, personal fees from Roam Insights, personal fees and non-financial support from United Network for Organ Sharing, personal fees and non-financial support from American Association of Allergy Asthma and Immunology, personal fees and non-financial support from University of Michigan, personal fees from Autodesk, personal fees and non-financial support from Regenstrief Institute, non-financial support from American Medical Association, personal fees and non-financial support from Precision Medicine World Conference, personal fees and non-financial support from University of Chicago, non-financial support from Mars, personal fees and non-financial support from Kneed Media, personal fees and non-financial support from Novartis, grants, personal fees and non-financial support from Janssen, personal fees and non-financial support from Detroit International Research and Education Foundation, personal fees and non-financial support from Siemens, personal fees and non-financial support from Georgetown University, personal fees and non-financial support from International Society for Advancement of Cytometry, personal fees and non-financial support from Stanford University, non-financial support from Microsoft, personal fees and non-financial support from Society for Prevention Research, personal fees and non-financial support from Washington University School of Medicine, non-financial support from American Society for Clinical Investigation, personal fees and non-financial support from Dana Farber Cancer Institute, personal fees, non-financial support and other from Mango Tree. A.L., M.I., L.R., and O.G. declare no competing interests.
