# Supplementary material for: Development and validation of a generalisable machine learning algorithm for identifying interstitial lung disease cohorts: a retrospective cohort study
Source: eClinicalMedicine. 2026 Feb 21;93:103790. doi: 10.1016/j.eclinm.2026.103790 (PMC12945527; doi:10.1016/j.eclinm.2026.103790)
Supplement: Supplement Table and Figures [file mmc1.pdf]

SUPPLEMENT

Supplement Table 1. Internal and External Comparison of Serial Model Performance

	PPV	Sensitivity	F1 Score	AUC
ILD Classification Algorithm	0.791	0.871	0.829	0.929
Modified ILD Classification Algorithm	0.719	0.820	0.766	0.847
Universal ILD Classifier (UCSF)	0.716	0.988	0.830	0.954
Universal ILD Classifier (External)*	0.667	0.974	0.792	0.956

*Average performance across three validation sites.

Supplement Figure 1. Distribution of Universal ILD Classifier Predicted Probabilities on General Population vs. Expert Annotations

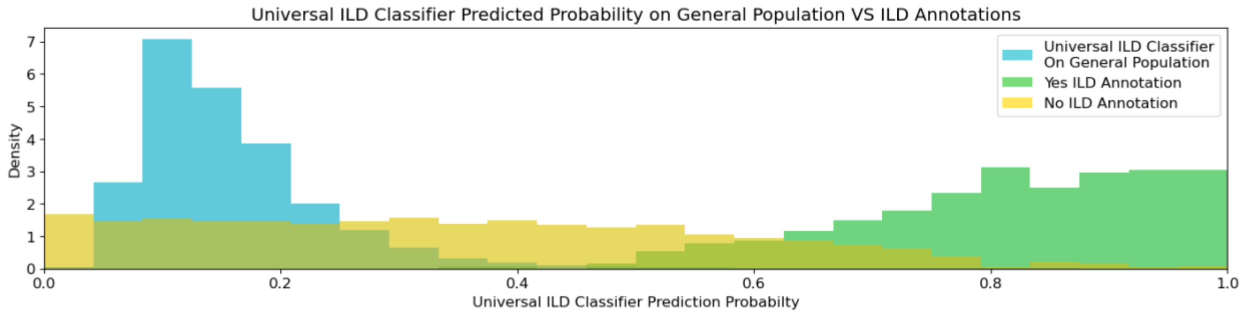

Distribution of ILD Classifier prediction probabilities on a general adult population shows clustering around 0.0-0.3, demonstrating that most adults have a low probability of ILD, as expected for a rare disease. Random sampling for outcome verification would therefore be unlikely to include patients with ILD and overestimate model performance. Alternatively, uniformally sampling across the range of probabilities from 0.0 to 1.0 enables for assessment of the model across the full range of possibilities and guarantees inclusion of positive ILD cases.

Supplement Figure 2.

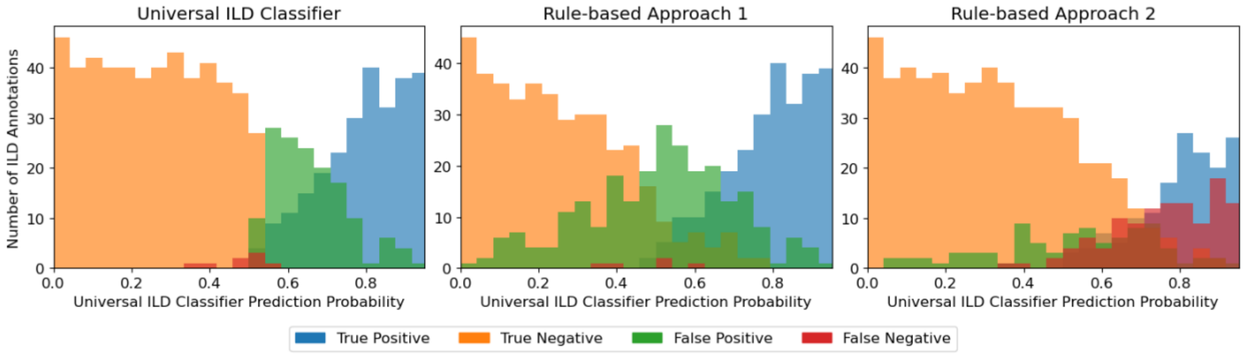

Plot of true positive, true negative, false positive, and false negative classifications for each of the three approaches, Universal ILD Classifier, Rule-based Approach 1, and Rule-based Approach 2. For both Rule-based approaches, misclassifications were made at all likelihoods of ILD, including false positives for individuals with low probability ILD and false negatives for patients with high probability ILD.
